# Supplementary material for: Detecting Central Auditory Processing Disorders in Awake Mice
Source: Brain Sci. 2023 Oct 31;13(11):1539. doi: 10.3390/brainsci13111539 (PMC10669832; doi:10.3390/brainsci13111539)
Supplement: Supplementary file 1 [file brainsci-13-01539-s001.zip › brainsci-2620168-supplementary.pdf]

Journal of
Clinical Medicine

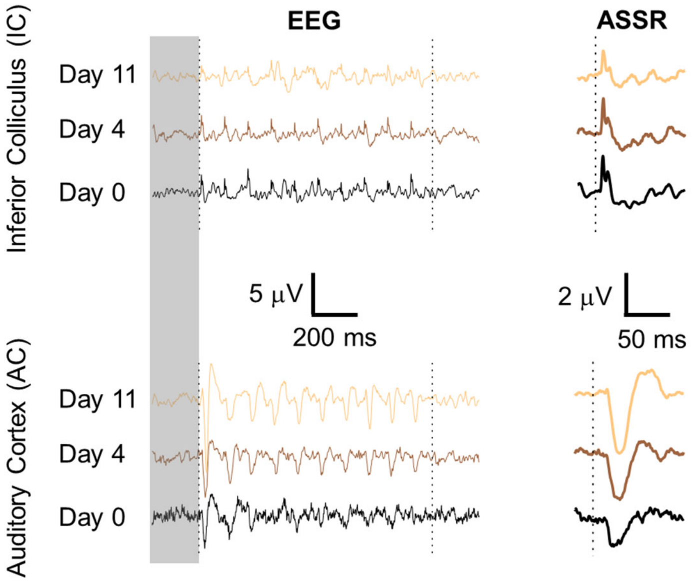

Supplementary Figure S1. Repeated recordings, over a period of 11 days, on the IC (top) and AC (bottom) of a single animal. Click trains at 10 Hz were used here. EEG response signals are shown on the left, with the ASSR response at 10 Hz, similar to that in Figure 1Ei, shown on the right. Despite the obvious variability of the EEG response, the ASSR response remained fairly stable over several days.

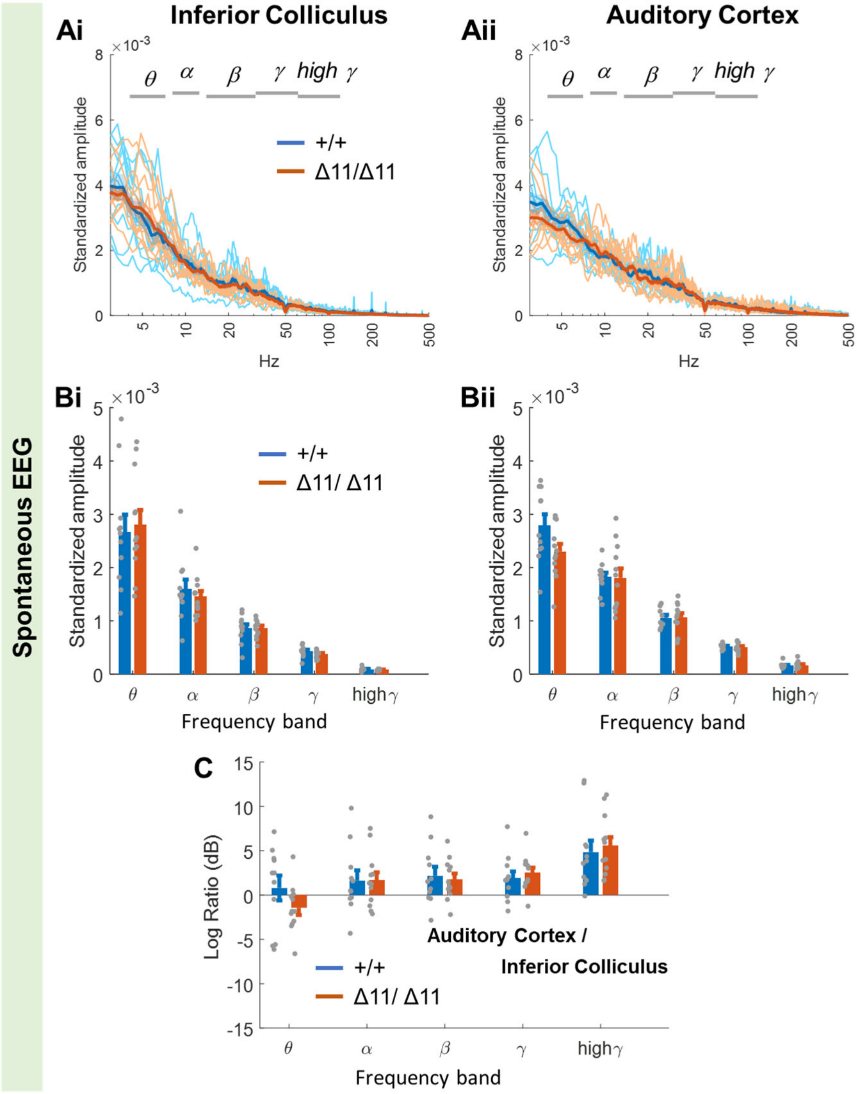

Supplementary Figure S2. Spontaneous EEG on the inferior colliculus (IC) and the auditory cortex (AC). A) Frequency spectrum based on standardized EEG signals in the Ai) IC and Aii) AC for control and mutant *Shank3*$^{\Delta 11/\Delta 11}$ mice. B) Amplitude spectrum averaged over classical frequency bands in the Bi) IC and Bii) AC and compared between control and mutant *Shank3*$^{\Delta 11/\Delta 11}$ mice. No significant difference was detected. C) Ratio of amplitude spectrum in the AC to amplitude spectrum in the IC, averaged over classical frequency bands.

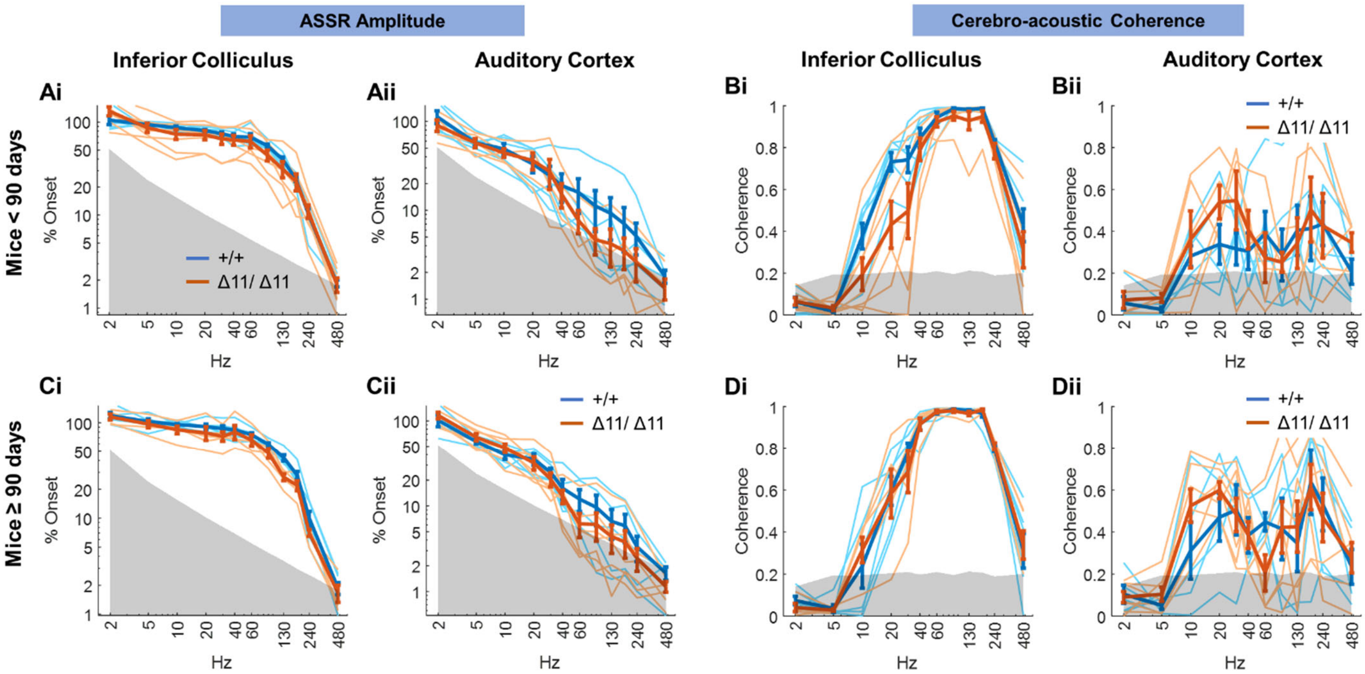

Supplementary Figure S3. Measurements of phase-locking of Fig. 2FG for animals younger and older than 90 days. A) ASSR amplitude in Ai) the IC and Aii) the AC relative to the onset response, for animals of age < 90 days at the time of recording. B) Same for animals of age ≥ 90 days at the time of recording. C) as for A) for cerebroacoustic coherence (CAC). D) as for B) for CAC. A-D) Thick lines indicate the mean values and thin lines are individual curves. The gray area corresponds to the 97.5th percentile of the ASSR calculated for simulated data (see methods). The error bar or the shaded area around the mean indicates the standard error of the mean.

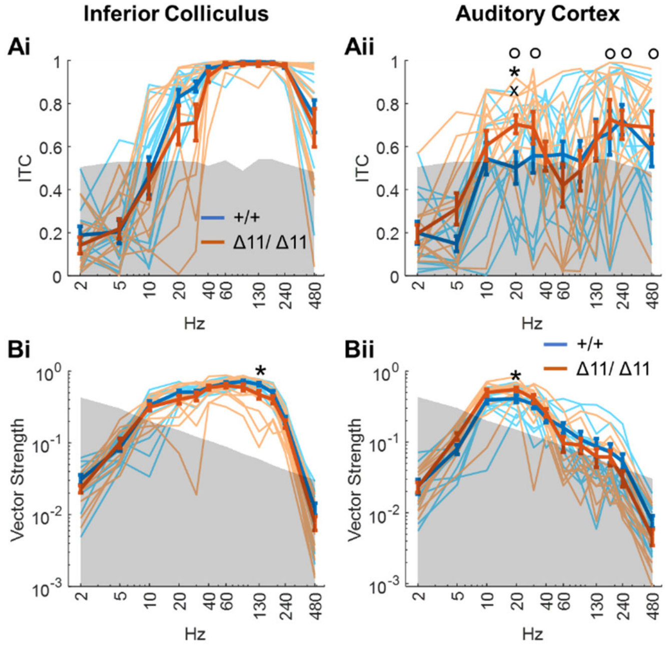

Supplementary Figure S4. Other measurements of phase-locking. A) Intertrial coherence (ITC) in Ai) the inferior colliculus (IC) and Bii) the auditory cortex (AC). B) Vector strength in B1) the IC and Bii) the AC. A-B) Thick lines indicate the mean values, whereas thin lines are individual curves. The gray area corresponds to the 97.5[th] percentile of the measurement calculated from simulated data (see methods). "*": *t*-test ctrl vs. mutant animals *p* value<0.05. "x": proportion test for significant measurements, *p* value<0.05. "o": *t*-test of a given rate vs. 60 Hz for mutant animals, *p* value<0.05. The error bar indicates the standard error of the mean.
